# Prognostic Structural Neural Markers of MRI in Response to Mechanical Thrombectomy for Basilar Artery Occlusion

**DOI:** 10.3389/fneur.2021.593914

**Published:** 2021-06-09

**Authors:** Chang Liu, Jia-Xin Song, Zhang-Bao Guo, Lu-Ming Chen, Chen-Hao Zhao, Wen-Jie Zi, Qing-Wu Yang

**Affiliations:** ^1^Department of Neurology, Xinqiao Hospital and The Second Affiliated Hospital, Army Medical University (Third Military Medical University), Chongqing, China; ^2^Department of Neurology, Wuhan No. 1 Hospital, Chongqing, China

**Keywords:** basilar artery occlusion, neural markers, MRI, mechanical thrombectomy, prognosis

## Abstract

**Objective:** Mechanical thrombectomy (MT) has been an effective first-line therapeutic strategy for ischemic stroke. With impairment characteristics separating it from anterior circulation stroke, we aimed to explore prognostic structural neural markers for basilar artery occlusion (BAO) after MT.

**Methods:** Fifty-four BAO patients with multi-modal magnetic resonance imaging at admission from the multicenter real-world designed BASILAR research were enrolled in this study. Features including volumes for cortical structures and subcortical regions, locations and volumes of infarctions, and white matter hyperintensity (WMH) volumes were recorded from all individuals. The impact features were identified using ANCOVA and logistic analysis. Another cohort (*n* = 21) was further recruited to verify the prognostic roles of screened prognostic structures.

**Results:** For the primary clinical outcome, decreased brainstem volume and total infarction volumes from mesencephalon and midbrain were significantly related to reduced 90-day modified Rankin score (mRS) after MT treatment. WMH volume, WMH grade, average cortex thickness, white matter volume, and gray matter volume did not exhibit a remarkable relationship with the prognosis of BAO. The increased left caudate volume was obviously associated with early symptomatic recovery after MT. The prognostic role of the ratio of pons and midbrain infarct volume in brainstem was further confirmed in another cohort with area under the curve (AUC) = 0.77.

**Conclusions:** This study was the first to provide comprehensive structural markers for the prognostic evaluation of BAO. The fully automatic and semiautomatic segmentation approaches in our study supported that the proportion of mesencephalon and midbrain infarct volume in brainstem was a crucial prognostic structural neural marker for BAO.

## Introduction

Accounting for only 1–4% of ischemic strokes, basilar artery occlusion (BAO) is a rare but devastating subtype of stroke, causing over 70% of deaths or a substantial part of severe disability in survivors (1), which has obtained abundant attention recently (2). In 2020, the results of the BEST randomized controlled trial and our real-world multicenter BASILAR research illustrated the improvement of mechanical thrombectomy (MT) for BAO (3, 4). However, despite this progress, the current understanding of the prognostic imaging markers of BAO in response to MT was limited, due to the challenges faced in performing multi-modal magnetic resonance imaging (MRI), which were caused by the rare prevalence of BAO and the low usage rate of MRI in a preoperative examination (2).

As an extremely lethal disease, BAO was characterized by lesions located subcortically with clinical manifestations significantly different from those of anterior circulation stroke (1). These impaired subcortical areas contained the brainstem, thalamus, and cerebellum, which were the essential components of conduction bundles and neural circuits, helping in the maintenance of multiple brain functions including consciousness maintenance, motor activities, and verbal response (5, 6). Although important physiological functions of these regions have been extensively explored, detailed effects of infarction locations and impairment volumes on the clinical outcome of BAO remained largely unclear (7, 8). In addition to lesion characteristics, multi-modal MRI provided much more comprehensive information according to previous literatures (9, 10). In T1-weighted imaging, the volumes for normal-appearing brain tissues were reported to predict the functional impairments in multiple neurodegenerative diseases, such as Alzheimer's disease and Parkinson's disease; however, none of the previous reports focused on their roles in BAO (8, 11). Moreover, the white matter hyperintensity (WMH) was a clinically important and highly heritable cerebrovascular phenotype on axial T2 fluid-attenuated inversion recovery (FLAIR), which was an essential indicator of neurological deterioration and was related to multiple clinical phenomena, including aging, dementia, and cognitive decline (12, 13). Although neurological findings of WMH were also linked to cerebral hypoperfusion and impaired white matter integrity, little attention has been paid to their characteristics in BAO (14).

Therefore, based on the largest cohort of BAO worldwide, constructed in BASILAR research, our study sought to explore the prognostic roles of neural markers in BAO patients treated with MT, by employing an advanced morphometry analysis for multi-modal MRI. This quantitative analysis would contribute to the establishment of a more comprehensive prognosis evaluation system for BAO after MT and promote the exploration of therapeutic targets.

## Materials and Methods

### Participants

In this *post-hoc* analysis, among all 647 BAO patients who underwent endovascular treatment in this multicenter real-world BASILAR research, 54 patients from different medical centers after MT with multi-modal MRI detection including FLAIR, diffusion-weighted imaging (DWI), and T1-weighted imaging at admission were enrolled in the training cohort, while another 21 patients with DWI at admission were recruited in the validation set. Our previous research has described the inclusion and exclusion criteria, as well as the baseline features of patients in detail (4). The analysis of clinical data and imaging material was approved by the BASILAR group. Written informed consent was obtained from all the patients or their legal representatives (4). Due to the much poorer prognosis of BAO than anterior circulation stroke, BAO patients with modified Rankin score (mRS) ≤ 3 on the 90th day after intervention were expected to achieve good recovery status, while subjects with mRS > 3 showed poor recovery, in accordance with recent literature (15, 16).

### Image Processing

To reduce the influence of different MRI examination equipment from different hospitals, semiautomatic or automatic algorithms and software verified on different platforms by numerous papers were used in this study (17–19).

Volumetric segmentation for T1-weighted imaging MRI slices was performed using an automatic FreeSurfer image analyzing software suite (http://surfer.nmr.mgh.harvard.edu) in the parallel arithmetic mode (http://www.gnu.org/software/parallel/). These processes included motion correction, removal of non-brain tissue using a hybrid watershed/surface deformation procedure, automated Talairach transformation, segmentation of the cortex, subcortical white matter and deep gray matter volumetric structures, and volume estimation (17).

For DWI, the 3D gross infarction volume was semiautomatically outlined by a continuous boundary-tracing algorithm based on an in-house segmentation script written in Matlab (The MathWorks, Inc., Natick, United States) to reduce user interaction and increase accuracy. The infarction areas in the PC-ASPECTS area, including the medulla, pontine, midbrain, and cerebellum, were isolated separately. In the testing cohort, impact prognostic structures identified in the training group were also delineated using DWI to evaluate their volumes manually. These impairments were identified by two experienced neuroradiologists and, in case of debate, were decided upon after discussion by a third neurologist. All clinical outcomes and patient information were blinded to the neuroradiologists (19).

In FLAIR slices, WMHs were segmented by placing a single seed within each visible WMH using 3D slicer software (https://www.slicer.org, Version 4.10.2). WMH borders were then adjusted by the users, as required, to correct errors such as mis-identification of the skull and the acute lesions in DWI slices. WMH volumes were quantified using the spatial dimensions of the voxels in each MRI slice. A consensus was considered if uncertainty existed (18).

### Statistical Methods

Continuous variables in demographic data and clinical features were compared using independent sample *t-*test or Mann–Whitney *U-*test. Categorical variables were compared by χ^2^ or Fisher's exact test, as appropriate. Descriptive statistics were expressed as mean (SD) or median (interquartile range) for continuous variables and as frequencies (percentages) for categorical variables. Each regional volume was analyzed by ANCOVA controlling for the total cranial brain volume, age, and sex (17). The relationship between screened differential regions and mRS, as well as early symptomatic recovery, indicated by decreased National Institutes of Health Stroke Scale (NIHSS), was investigated using logistic regression analysis. A backward selection procedure was chosen for the multiple logistic regression. Discrimination was measured by the area under the curve (AUC) in both testing and validation cohorts. All analyses were performed using the R platform (https://www.r-project.org) with an in-house script. All reported *p*-values were two-tailed. The level of significance was set at *p* < 0.05.

## Results

### Demographic Characteristics

The baseline clinical features of the recruited patients are shown in Table 1. Among them, 21 patients showed favorable clinical outcomes of 90-day mRS after the intervention. Between these subjects with differential clinical outcomes, patients with poor clinical outcome exhibited a decline in the initial NIHSS [29 (14.00–34.00) vs. 16 (8.00–22.50), *p* = 0.03] and NIHSS at 24 h [35.00 (30.00–35.50) vs. 10.00 (4.50–19.50), *p* < 0.01] after MT. However, no significant alterations in imaging time, intervention time, systolic BP, diastolic BP, sex, or age (all *p* > 0.05) were found among them.

**Table 1:** Demographic characteristics of enrolled patients.

	**mRS ≤ 3 (*N* = 21)**	**mRS > 3 (*N* = 33)**	***p*-value**
**Demographic information**			
Age (years)	58.38 ± 9.14	63.42 ± 9.47	0.07
Female sex no. (%)	4 (19.05%)	6 (18.18%)	1.00
Premorbid mRS, 0	18 (85.71%)	28 (84.84%)	1.00
**Treatment information**			
Initial NIHSS score	16.00 (8.00–22.50)	29.00 (14.00–34.00)	0.03^*^
PC ASPECTS score (DWI)	8.00 (6.50–9.00)	8.00 (7.00–9.00)	0.52
Recanalization time (min)	430.50 (277.30–1145.00)	500.00 (328.50–700.00)	0.84
Imaging time (min)	169.50 (64.75–649.00)	181.00 (30.00–273.00)	0.39
NIHSS score at 24 h after MT	10.00 (4.50–19.50)	35.00 (30.00–35.50)	0.00^*^
mRS at the 90th day after MT	2 (1–3)	5 (4–6)	0.00^*^
**Stroke information**			
Hypertension no. (%)	12 (57.14%)	22 (66.67%)	0.57
Diabetes mellitus no. (%)	3 (14.29%)	5 (15.63%)	1.00
Hyperlipidemia no. (%)	9 (42.86%)	12 (37.50%)	0.77
Atrial fibrillation no. (%)	6 (18.75%)	2 (9.52%)	0.46
Smoking no. (%)	13 (40.61%)	8 (38.09%)	1.00
Pre-stroke antithrombotic use no. (%)	4 (19.05%)	10 (32.26%)	0.35
Pulmonary infection no. (%)	14 (66.67)	29 (87.88%)	0.09
Posterior circulation collateral score (BATMAN)	5 (3.506.50)	5 (3.006.00)	0.39
TOAST classification			1.00
Large artery atherosclerosis	4	6	
Cardioembolism	17	27	
**Laboratory information**			
White blood cells (per μl)	10.48 ± 4.31	11.43 ± 3.78	0.41
Hemoglobin (g/dl)	139.75 ± 14.55	140.28 ± 20.93	0.92
Initial glucose (mg/dl)	7.21 ± 3.20	8.32 ± 2.81	0.21
HbA1c (%)	6.66 ± 1.23	6.54 ± 1.53	0.88
Total cholesterol (mg/dl)	5.18 ± 1.52	5.16 ± 1.19	0.97
HDL cholesterol (mg/dl)	1.31 ± 0.32	1.27 ± 0.35	0.72
Triglycerides (mg/dl)	1.54 ± 1.1	1.67 ± 1.05	0.70
LDL cholesterol (mg/dl)	2.94 ± 1.29	3.25 ± 1.04	0.41
Systolic BP (mm Hg)	144.55 ± 24.5	152.7 ± 24.09	0.24
Diastolic BP (mm Hg)	86.8 ± 15.78	91.73 ± 19.97	0.35

*NIHSS, National Institutes of Health Stroke Scale Score; mRS, modified Rankin score; TOAST, Trial of ORG 10172 in Acute Stroke Treatment; LDL, low-density lipoprotein; LDL, high-density lipoprotein; BP, blood pressure; DWI, diffusion-weighted imaging; MT, mechanical thrombectomy; HbA1c, glycated hemoglobin*.

* *p < 0.05*.

### Predictors of Neural Markers for Modified Rankin Score Level

As shown in Table 2, ANCOVA revealed that following variables were significantly associated with poor outcomes: volume of midbrain and pontine infarction (MPI, 836.03 ± 850.46 vs. 412.14 ± 563.74 mm^3^, *p* = 0.04), total infarction volume (880.42 ± 936.27 mm^3^ vs. 414.38 ± 562.99 mm^3^, *p* = 0.04), the ratio of MPI in brainstem [RMPI 3.31% (1.55–5.49%) vs. 1.28% (0.00–1.74%), *p* = 0.04], brainstem volume (20497.3 ± 2741.62 mm^3^ vs. 19516.61 ± 2254.73 mm^3^, *p* = 0.02) and left caudate volume (2749.63 ± 528.09 mm^3^ vs. 3030.33 ± 421.15 mm^3^, *p* = 0.03). Other variables did not exhibit significant associations with poor outcomes (all *p* > 0.05). In multivariate logistic regression analysis using the backward selection method, the following variables remained independent predictors of 90-day poor outcome: RMPI (adjusted OR 1.367; 95% CI: 1.061–1.861; *p* = 0.03) and baseline NIHSS (adjusted OR 1.086; 95% CI: 1.017–1.170; *p* = 0.02) (Table 3).

**Table 2:** Predictors of neural markers for mRS level.

**Characteristics and outcomes**	**Unfavorable outcome**	**Favorable outcome**	***p*-value**
Infarction volumes (mm^3^)			
Medulla	44.39 ± 185.69	18.00 ± 72.45	0.71
Mesencephalon	734.85 ± 873.64	348.67 ± 481.39	0.07
Midbrain	101.18 ± 213.59	44.75 ± 129.61	0.11
MPI	836.03 ± 850.46	412.14 ± 563.74	0.04^*^
Total infarctions in brainstem	880.42 ± 936.27	414.38 ± 562.99	0.04^*^
Left cerebellum	3314.94 ± 10351.33	1279.1 ± 4381.47	0.15
Right cerebellum	1984.09 ± 5750.58	732.7 ± 1387.59	0.30
Left thalamus	82.39 ± 256.12	44.95 ± 175.84	0.46
Right thalamus	106.67 ± 234.65	36.15 ± 88.92	0.10
Left hemisphere	635.58 ± 1959.07	2185.15 ± 8020.8	0.29
Right hemisphere	734.73 ± 2414.06	1800.95 ± 4680.18	0.68
Total infarctions	7738.82 ± 13387.39	6476.75 ± 10130.2	0.31
RMPI (%)	3.31 (1.55–5.49)	1.28 (0.00–1.74)	0.04^*^
Ratio of brainstem infarction in brainstem (%)	3.31 (1.55–5.91)	1.56 (0.00–2.38)	0.06
WMH			
WMH volume (mm^3^)	3,349 (801–4,707)	1,067 (383.5–2,847)	0.19
Fazekas grade no. (%)			0.32
1	12 (36.36%)	12 (57.14%)	
2	17 (51.52%)	7 (33.33%)	
3	4 (12.12%)	2 (9.52%)	
Brain regions (mm^3^)			
Left lateral ventricle	14829.53 ± 7924.46	17199.86 ± 9285.28	0.09
Left cerebellum white matter	16063.05 ± 4537.84	16626.58 ± 3576.11	0.97
Left cerebellum cortex	45846.42 ± 8049.27	45745.42 ± 5562.31	0.69
Left thalamus proper	6562.46 ± 1370.24	6737.89 ± 1285.84	0.87
Left caudate	2749.63 ± 528.09	3030.33 ± 421.15	0.03^*^
Left putamen	3844.15 ± 652.34	3893.1 ± 495.14	0.92
Left pallidum	1618.39 ± 318.19	1658.06 ± 267.77	0.78
3rd ventricle	1726.4 ± 628.86	1735.07 ± 530.89	0.47
4th ventricle	1819.07 ± 544.99	1897.68 ± 586.53	0.35
Brain stem	20497.3 ± 2741.62	19516.61 ± 2254.73	0.02^*^
Left hippocampus	4095.19 ± 854.95	4123.75 ± 777.59	0.83
Left amygdala	1415.3 ± 249.88	1449.52 ± 270.01	0.99
Left accumbens area	278 ± 78.73	330.79 ± 83.36	0.18
Right lateral ventricle	12454.01 ± 5499.8	14604.84 ± 7365.23	0.06
Right cerebellum white matter	14215.7 ± 4021.48	14769.07 ± 2788.85	0.66
Right cerebellum cortex	46070.25 ± 7718.45	46627.56 ± 4889.12	0.74
Right thalamus proper	6708.68 ± 1199.69	6466.9 ± 1221.64	0.14
Right caudate	2781.4 ± 605.44	2948.57 ± 601.07	0.34
Right putamen	3888.56 ± 561.56	3721.23 ± 639.33	0.13
Right pallidum	1677.29 ± 339.94	1623.52 ± 306.95	0.69
Right hippocampus	4295.85 ± 992.84	4545.77 ± 762.03	0.37
Right amygdala	1591.58 ± 285.54	1667.46 ± 231.23	0.53
Right accumbens area	324.29 ± 112.22	344.66 ± 71.78	0.88
Optic chiasm	105.88 ± 55.31	117.7 ± 42.91	0.18
CC posterior	1085.44 ± 316.77	994.84 ± 344.35	0.45
CC mid-posterior	824.95 ± 277.03	755.36 ± 223.31	0.39
CC central	862.7 ± 294.37	789.17 ± 336.4	0.45
CC mid-anterior	801.75 ± 310.92	704.32 ± 315.76	0.36
CC anterior	931.57 ± 204.13	841.06 ± 364.17	0.42
Left cortex thickness	2.38 ± 0.23	2.39 ± 0.24	0.79
Left white surface total area	63970.28 ± 8507.82	62360.77 ± 4934.71	0.37
Right cortex thickness	2.36 ± 0.23	2.34 ± 0.23	0.59
Right white surface total area	64122.3 ± 10342.73	61648.35 ± 6479.9	0.15
Left hemisphere cortical gray matter volume	163471.89 ± 25611.42	160002.86 ± 26686.61	0.47
Right hemisphere cortical gray matter volume	163405.88 ± 27872.54	156468.72 ± 26313.51	0.29
Total cortical gray matter volume	327196.49 ± 53388.14	311070.24 ± 66682.86	0.29
Subcortical gray matter volume	52085.03 ± 6317.64	52536.25 ± 6075.36	0.73
Total gray matter volume	476496.63 ± 54712.06	466838.56 ± 42908.06	0.02^*^7

*MPI, infarction volume in midbrain and pons; RMPI, the ratio for infarction volume in midbrain and pons; mRS, modified Rankin score; WMH, white matter hyperintensity*.

* *p < 0.05*.

**Table 3:** Univariate and multivariate logical analyses for clinical outcome predictors.

	**Univariate logistic regression**			**Multivariate logical analysis**		
	**Crude OR**	**95% CI**	***p*-value**	**Adjusted OR^a^**	**95% CI**	***p*-value**
Initial NIHSS	1.061	1.007–1.124	0.03^*^	1.086	1.020–1.170	0.02^*^
Caudate (mm^3^)	0.999	0.997–0.999	0.05^*^	—		
MPI (mm^3^)	1.001	1.000–1.002	0.06	—		
RMPI (%)	1.270	1.028–1.681	0.05^*^	1.367	1.061–1.861	0.03^*^
Total infarctions in brainstem (mm^3^)	1.001	1.000–1.002	0.06	—		
Brainstem (mm^3^)	1.000	0.999–1.000	0.14	—		

*NIHSS, National Institutes of Health Stroke Scale Score; MPI, infarction volume in midbrain and pons; RMPI, the ratio for infarction volume in midbrain and pons; mRS, modified Rankin score; WMH, white matter hyperintensity*.

a *Adjusted for age, sex, and total cerebral volume*.

* *p < 0.05*.

### Association of Neural Imaging Measurements With Early Recovery After Mechanical Thrombectomy

Table 4 illustrates the association of the essential imaging markers identified above with the early improvement of NIHSS. After age, sex, and brain volume were adjusted, a positive relationship between left caudate volume and decreased NIHSS at 24 h post-operative was found (*p* = 0.01). However, we did not detect other significant neural imaging markers for the early improvement of clinical manifestations (all *p* > 0.05).

**Table 4:** Association of neural imaging measurements with early recovery after MT.

**Item**	**None recovery**	**Early recovery**	***p*-value**
Caudate (mm^3^)	2735.69 ± 469.73	3202.02 ± 431.27	0.01^*^
MPI (mm^3^)	769.43 ± 836.46	390.5 ± 482.85	0.13
RMPI (%)	2.47 (0.94–4.71)	1.42 (0.00–2.58)	0.20
Total infarctions in	798.33 ± 907.5	415.93 ± 522.06	0.17
brainstem (mm^3^)			
Brainstem (mm^3^)	20145.59 ± 2631.56	19888.34 ± 2557.29	0.21

*MT, mechanical thrombectomy; MPI, infarction volume in midbrain and pons; RMPI, the ratio for infarction volume in midbrain and pons*.

* *p < 0.05*.

### Validation of RMPI in Another Cohort

**Figure 1:**
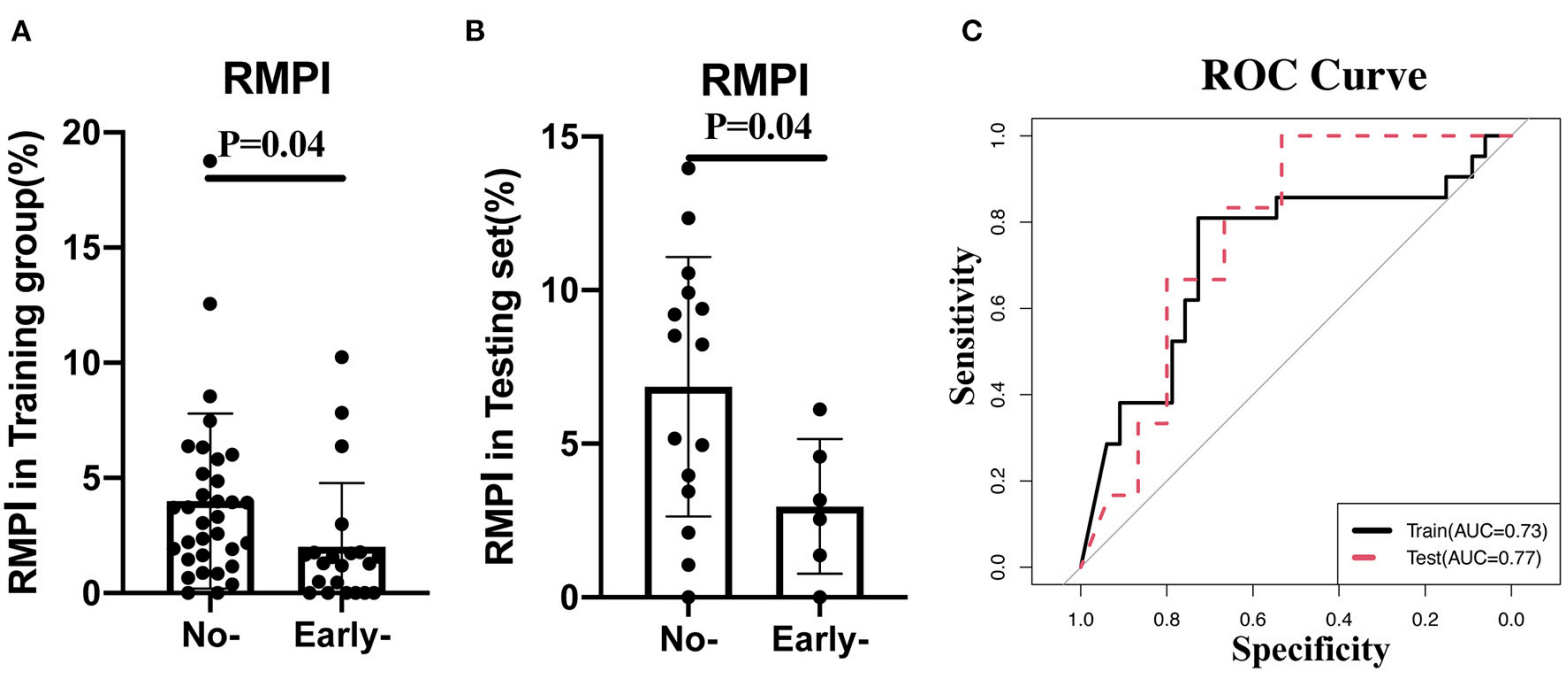
Validation of RMPI in the testing cohort. **(A,B)** The expression pattern of RMPI in the training and testing groups. **(C)** RMPI achieved discriminative power in the training group and testing set. RMPI, the ratio for infarction volume in midbrain and pons.

Given the essential role of RMPI in predicting clinical outcomes, we further validated its predictive role in another cohort, whose clinical manifestations are illustrated in Supplementary Table 1. As shown in Figures 1A,B, in both training and validation cohorts, RMPI levels were obviously elevated in those with poor clinical outcomes based on univariate analysis (training group: *p* = 0.04; testing set: 6.86 ± 4.23% vs. 2.96 ± 2.19%, *p* = 0.04). In addition, RMPI achieved discriminative power with the AUC = 0.73 (95% CI: 0.578–0.876) and AUC = 0.77 (95% CI: 0.567–0.977) in the training group and testing sets, respectively (Figure 1C).

## Discussion

To our knowledge, this is the first multicenter MRI study to comprehensively evaluate the prognostic value of neural markers for BAO patients in response to MT treatment. After screening these essential factors, including infarction volumes, impairment locations, WMH characteristics, and brain region volumes, our results indicated that a reduction in the ratio of midbrain and pons infarction volume in brainstem was an independent predictor of clinical outcome after MT intervention. Our findings also revealed that increased caudate volume was positively related to early recovery after stroke. These results promote the understanding of MT preoperative evaluation and highlight crucial brain structures in determining the clinical outcome of BAO.

According to the literatures on anatomy, among all impaired brain regions, after occlusion of the basilar artery, the brainstem was a crucial function center that aided in the maintenance of consciousness and physiological activities, including balance control, coordinated movement, hearing, speech, eye movement, and swallowing. Consistent with previous reports, extensive baseline brainstem impairment in BAO patients implied a remarkably increased rate of futile recanalization (20). However, our results further provided quantitative volume information for infarcts in the midbrain, pons, and medulla, whereas positional analysis from some previous studies might amplify the impacts of small infarcts on the clinical outcome (21, 22). Recent research has shown that pontine injury was associated with an extremely poor prognosis (23). Our results did not find the prognostic roles of pons but detected that the total volume of pontine and midbrain injury was predictive. With numerous conduction systems crossing both pons and midbrain, simultaneous blockage at different levels could result in much less residual conduction than the pons alone, leading to much worse clinical outcome (24). As for the relative relationship between infarcts and brainstem, the relative ratio of MPI might represent the degree of damage to the nuclei and tracts in the brainstem, such as corticospinal tract and corticobulbar tract (25). A recent report by Yoon focused on the cross-sectionally extensive degree in pons (26). Cho sectioned three typical MRI slices of brainstem and scored infarctions based on these new sections (25). Lu also found that the maximum length of infarction in brainstem could predict the neurologic deterioration (27). Compared with the results of these studies, our results provide more accurate volume information through quantitative or semi-quantitative algorithms, which reduced the interference of artificial measurement, and demonstrated that the volume ratio for MPI volume in brainstem was an independent prognostic factor for BAO. Since that the spatial distribution of nuclear masses in brainstem was multilevel, using a single section might be insufficient to reflect the degree of damage, while our volume ratio provided 3D spatial information that helped to assess the extent of damage more systematically (24). Moreover, the compression of brainstem caused by edema in cerebellar or other structures was an important reason for the poor prognosis of patients. Therefore, taking brainstem volume into account helped to analyze the influence of other tissues on the brainstem, which could further effectively improve the accuracy of our model (28).

The predominant connectivity with the thalamus, frontal area and cerebellum, and caudate nuclei was a key associative structure in networks that controlled the motor, behavior, and execution functions. In particular, they aided in maintaining body and limb posture, as well as controlling approach-attachment behaviors (29, 30). In line with a previous study with a large sample of patients with anterior circulation stroke, preservation of the caudate nucleus was positively related to the favorable clinical outcome of stroke (21). Recent studies also reported decreased caudate volume in those who failed to maintain a steady gait. In addition, a positive relationship was found between the atrophic left caudate nucleus and impaired language or speech abilities, which were important indicators for the postoperative outcome evaluations (31, 32). The disruption or reduction of functional connectivity caused by atrophy of the caudate, such as cortex-neostriatum loop and fronto-subcortical circuits that mediated executive cognition of organizing, sequencing, and anticipating future consequences, might be the main cause for the poor prognosis after MT (33, 34). Blood supply was a key issue in regulating the volumes of these nuclei, in addition to genetic heterogeneities. The decreased size of the nucleus implied cerebrovascular insufficiency and poor vascular conditions. This, in turn, would lead to vulnerable nuclei and poor recovery conditions with insufficient reperfusion after stroke (29, 35). In addition, as these were key components of neural circuits, damaged links among these circuits caused by pre-stroke injury or insufficient perfusion often led to negative feedback in the hub nucleus such as the caudate area and decreased their volumes (36).

This study has several limitations. First, the relatively small sample size might have downplayed the significance of influential nucleus, even though our cohort was constructed in a multicenter setting and validated in another cohort. We will re-evaluate prognostic roles of these brain regions in another clinical trial for BAO patients conducted by our groups. Second, it was difficult to fully understand the biological value of these impact nuclei and impairments due to its observational experimental design and data-driven approach. Interventional animal experiments, functional MRI, and prospective studies are needed to further verify these results and explore their detailed functions. Third, based on this automated algorithm, it took ~10 h per person to calculate the volumes of all brain structures. In clinical applications, artificial intelligence software may be needed to obtain essential predictive structures more accurately and quickly.

In conclusion, this multicenter study demonstrated the significantly increased caudate volumes and reduced infarction volumes from pons and midbrains in patients with favorable clinical outcome after MT. Given the detailed relationship between the brain morphology and clinical manifestations in NIHSS and 90-day mRS, our results suggest the potential of this morphological index in the recovery of stroke and that it might complement the MT preoperative evaluation criteria.

## Data Availability Statement

The original contributions presented in the study are included in the article/Supplementary Material, further inquiries can be directed to the corresponding author/s.

## Ethics Statement

The studies involving human participants were reviewed and approved by Xinqiao Hospital and The Second Affiliated Hospital, Army Medical University. The patients/participants provided their written informed consent to participate in this study.

## Author Contributions

L-MC and J-XS wrote the manuscript. C-HZ and Z-BG contributed to the writing process. L-MC, W-JZ, and Q-WY analyzed and interpreted the data and prepared the tables and figures. W-JZ, C-HZ, and Z-BG acquired the data. W-JZ and Q-WY additionally contributed to the conception and the design of the study. All the co-authors read and revised the article.

## Conflict of Interest

The authors declare that the research was conducted in the absence of any commercial or financial relationships that could be construed as a potential conflict of interest.
