# Supplementary material for: Prognostic Structural Neural Markers of MRI in Response to Mechanical Thrombectomy for Basilar Artery Occlusion
Source: Front Neurol. 2021 Jun 9;12:593914. doi: 10.3389/fneur.2021.593914 (PMC8220209; doi:10.3389/fneur.2021.593914)
Supplement: Supplementary file 1 [file Table_1.DOCX]

|  | **Testing group**  **(N=21)** |
| --- | --- |
| **Demographic information** |  |
| Age(year) | 57.37±10.88 |
| male sex no.(%) | 12(63.16%) |
| Premorbid mRS, 0 | 21(100.00%) |
|  |  |
| **Treatment information** |  |
| Initial NIHSS score | 23.00(20.00-32.00) |
| PC ASPECTS score (DWI) | 8.00(7.00-9.00) |
| NIHSS score at 24 hours after MT | 27.00(17.00-36.00) |
| mRS at 90^th^ day after MT | 5(3-5) |

Supplemental Table 1: Clinical manifestations for Testing group
